# Dementia Prevalence, Comorbidities, and Lifestyle Among Jatinangor Elders

**DOI:** 10.3389/fneur.2021.643480

**Published:** 2021-07-23

**Authors:** Paulus Anam Ong, Febby Rosa Annisafitrie, Novita Purnamasari, Chandra Calista, Noveline Sagita, Yulia Sofiatin, Yustiani Dikot

**Affiliations:** ^1^Department of Neurology, Hasan Sadikin Hospital, Universitas Padjadjaran, Bandung, Indonesia; ^2^Department of Neurology, Immanuel Hospital, Maranatha Christian University, Bandung, Indonesia; ^3^Department of Public Health, Faculty of Medicine, Universitas Padjadjaran, Bandung, Indonesia; ^4^Department of Neurology, Faculty of Medicine, Achmad Yani University, Bandung, Indonesia

**Keywords:** dementia, LMIC, lifestyle, prevalence Jatinangor Indonesia, comorbidities

## Abstract

**Introduction:** Research on dementia prevalence and the potentially related risk factors from Indonesia is scarce. We sought to identify the prevalence of dementia, health risk factors, and lifestyle in Jatinangor elders.

**Methods:** A total of 686 participants completed questionnaires on lifestyle, health risk factors, and cognitive and functional tests from September 2013 to December 2013. We determined the prevalence of dementia; and the associations between health, leisure activities, dietary pattern, and dementia were analyzed using logistic regression.

**Results:** The prevalence of dementia was 29.15%. The risk factors differed between age groups. Those aged 60–74 years and who have a lower education level, lower occupational attainment, and less active intellectual and recreational activities were associated with higher dementia risk. Those aged > 75 years living in a rural area and who take less fruit were associated with a higher risk of dementia.

**Conclusions:** The prevalence of dementia in Jatinangor is high. The identified modifiable risk factors are a potential target for intervention and valuable for designing public health policies.

## Introduction

An increase in people living with dementia (PWD) is one of the inevitable aging population consequences. Worldwide, there are ~50 million PWD in 2015, and this number is projected to increase to 152 million by 2050, rising particularly in low-income and middle-income countries (LMICs), where approximately two-thirds of PWD live (1). Like other countries, Indonesia was estimated to have a rapid increase of PWD, from 1.2 million in 2015 to 1.9 million in 2030, and this figure will reach 3.9 million in 2050 (2). The large number of PWD will cause serious healthcare and socioeconomic impacts if not anticipated.

Currently, there are no disease-modifying agents for dementia. Therefore, implementing evidence-based preventive strategies can mitigate the socioeconomic impact of dementia. Evidence of decline in the prevalence of dementia in high-income countries (HICs) indicated that dementia might, at least partially, be preventable by reducing the prevalent risk factors (3, 4). Moreover, increasing evidence suggests that lifestyle activities that optimize the use of compensatory cognitive strategies (5) and healthy dietary patterns might help preserve cognition in late life (6). A recent scientific paper was even more optimistic, targeting about 40% of dementia prevention by managing risk factors throughout the life span (7).

Finding from HICs may not be necessarily applicable directly in LMICs, the region with different sociodemographic and risk factors. Therefore, prevention programs that focus on local contexts and modifiable risk factors are needed to design effective interventions and appropriate public health policies. It is especially true for Indonesia, a middle-income country with large numbers of elders and limited healthcare resources, to apply nationwide established preventive measures. The present study sought to identify the modifiable risk factors for dementia, focusing on sociodemographic, health, and lifestyle risk factors, including leisure activities and dietary patterns.

## Methods

### Setting and Study Design

We used a cross-sectional study design and collected information from elders aged ≥ 60 years and their caregivers between September 2013 and December 2013 to examine the prevalence and determinants of dementia. The data collection consisted of sociodemographic characteristics (age, gender, education, living area, and marital status), socioeconomic status (best occupation and monthly income), health factors [hypertension, cholesterol, diabetes, body mass index (BMI), and depression], lifestyle including leisure activities (cognitive, social, recreational, physical activities), and dietary pattern, as well as cognitive and functional status assessments. All data were obtained in one seat by 15 trained, third-year medical school students and five neurology residents for field neurological examination consultation. Blood samples were drawn on another day. All participants gave written consent. The Medical Ethics Committee of Universitas Padjadjaran Bandung, Indonesia, approved this study protocol.

### Participants

This epidemiology study was done in the Sumedang Regency of West Java province, the most densely populated province in Indonesia. We selected Jatinangor from the total 22 districts as the representative of the Sumedang Regency, considering its accessibility from Universitas Padjadjaran. Jatinangor is located on the slope of Manglayang Mountain. Most of the villagers worked as farmers, employees in the plantation, or civil servants and had a total population of 113,913 people (8). Villages Cilayung and Cipacing were selected to represent the 12 villages, based on the village's status, such as self-made and self-sufficiency villages, respectively. A self-sufficiency village (suburban) has higher welfare, a higher proportion of professional vs. labor, and more self-employment workers than a self-made village (rural area). Cilayung village was selected from seven self-made villages, and Cipacing village was selected from a total of five self-sufficiency villages by stratified random sampling based on data from the sub-village registry.

**Figure 1:**
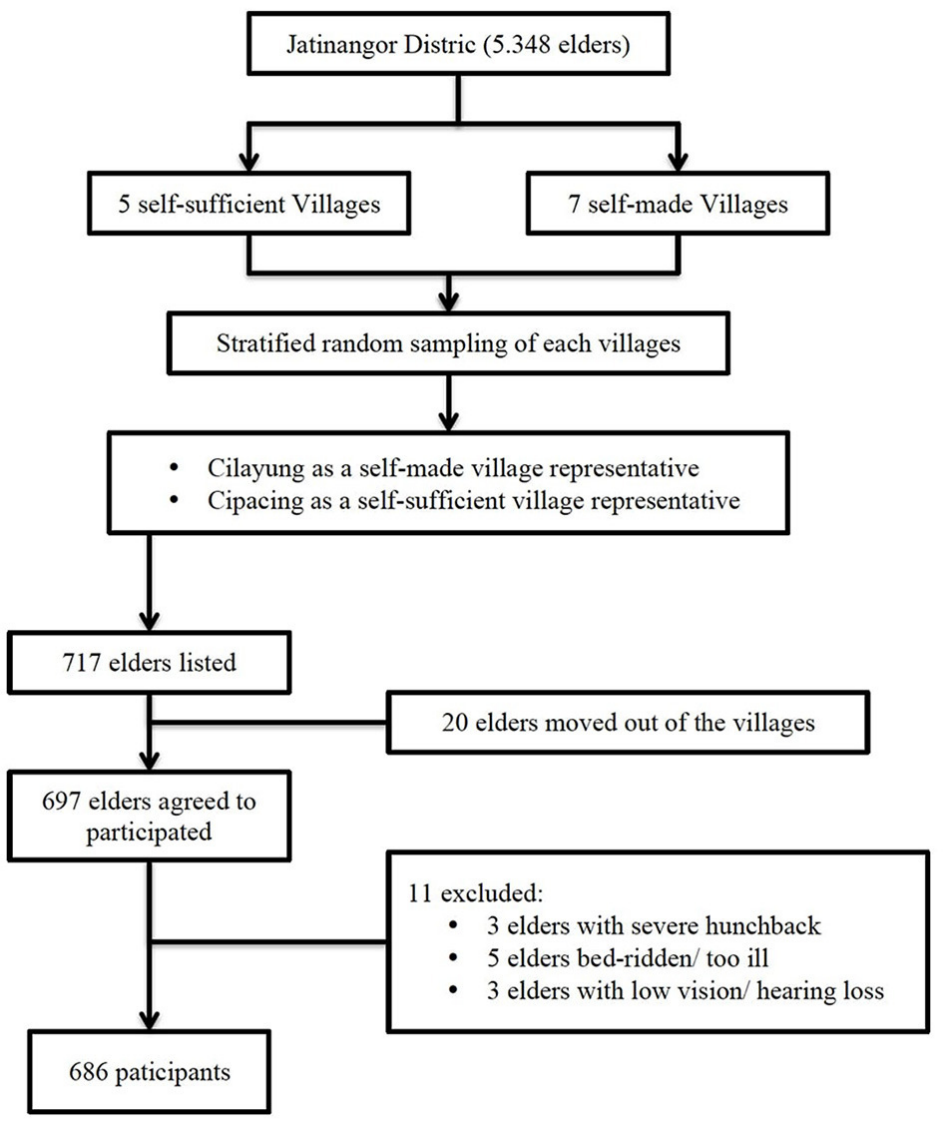
Flowchart describing flow of the participant recruitment in the study.

We involved all families with an elder (age ≥ 60 years) listed in the cadres' registration from the two selected villages. Meetings with local cadres were held according to village meeting schedules. Meetings with elders and their caregivers, explaining our research aims, participation, consequences, and ethical issues, and their willingness to participate was listed in coordination with cadres. Written consent was obtained from the elders or caregivers on the day of the interview commencement. From the total of 717 elders listed, 20 elders moved out of the villages, and the remained 697 elders agreed to participate (97.2% participation rate). Eleven elders were excluded, including three persons with a severe hunchback, whose BMI could not be measured; five bedridden elders; and three other elders with severe low vision and hearing loss. In this research, we obtained 686 participants. The number of participants exceeded the minimum sampling requirement determined with Slovin's formula, *n* = *N*/[1 + *N* (e)^2^] (9, 10), where *n* is the sample size, *N* is the population size, and e is the margin error. In this study, *N* = 5,348 (population aged ≥ 60 years in Jatinangor), e = 0.08, and the minimum number of participants *n* = 5,348/[1 + 5,348 (0.05)^2^] = 372 participants. Refer to Figure 1 for the detailed participant recruitment flowchart.

### Measures

We applied the National Institute on Aging/Alzheimer's Association (NIA-AA) criteria for dementia of all-cause, which requires a severe cognitive decline complained by an individual or a reliable informant, confirmed with an objective cognitive test, which interferes with daily living activities in the absence of delirium or other mental disorders (11). We used the Abbreviated Mental Test (AMT) (12), considering it was brief, used successfully in an extensive stroke cohort registry, and performed well in our local studies (13). A study done in Singapore reported no differences in diagnostic performance regarding the area under the curve between the Mini-Mental State Examination (MMSE) and AMT in diagnosing dementia among elders in Singapore. However, they found a trend of better diagnostic performance of AMT in patients with lower educational levels (0–6 years) and MMSE for those with higher levels of education (14). Therefore, considering most participants' low education level in our study population, we decided to use AMT for dementia cognitive screening with the cutoff score based on age category and education level as suggested by the previous study (14). For participants aged 60–74 years, we used a cutoff score of 7/8 for those with <6 years of education and a score of 8/9 for those with >6 years of education. For participants aged >75, we used a cutoff score of 5/6 for those with <6 years of education and a score of 8/9 for those with education >6 years (15). The AD8 was used to confirm the decline of cognitive function reported by an informant or caregiver, with a score ≥ 2 indicating cognitive impairment (14). We used the Katz Activities of Daily Living (16) and Lawton Instrumental Activities of Daily Living to assess functional declines (17).

### Health Factors

Hypertension was defined as a blood pressure ≥ 140/90 mmHg measured at the upper arm. Depression was diagnosed using the Geriatric Depression Scale (GDS), with a cutoff score of ≥9 indicating depression (18). Diagnosis of diabetes mellitus was made if the fasting plasma glucose is 126 mg/dl or higher. Dyslipidemias was considered high risk if triglyceride ≥ 200 mg/dl and/or high-density lipoprotein (HDL) cholesterol level <40 mg/dl. Triglyceride and HDL were used as indicators of dyslipidemia, as they are part of a metabolic syndrome and cardio-metabolic risk factors (19, 20). BMI characteristic was based on WHO classification.

### Lifestyle Factors

We adapted the classification of late-life leisure activities validated among the elderly in Hong Kong (21) used in CLASSA, a study coordinated by the Asian Society Against Dementia (ASAD). Leisure activities were categorized into physical, intellectual, social, and recreational categories. The participation of each activity from a category was summed and considered as a category activity. Participants were considered less active in a category if the activity of the category was below the mean of the total sample category activity, and vice versa with active participants in a category.

We used the food frequency questionnaire to examine dietary patterns, which consisted of seven categories of frequency: never or less than once/month, sometimes one to three times/month, once/week, three to four times/week, once/day, twice/day, and three times/day. Accompanied by their caregivers, subjects were asked to report their intake frequency on standard dietary, categorized as protein (eggs, meat, fish, and beans), carbohydrate (rice, noodles, bread, tubers, and flour), fruits, and vegetables. We also assessed local customary food, such as tofu/tempeh, instant noodles, and salted fish. A food intake was categorized as “non-frequent” if the frequency is below the total sample's median frequency and vice versa with active participants in a category.

### Data Analyses

We use descriptive statistics to compare the baseline characteristics of participants with and without dementia. Missing data were found in risk factor variables, 0–14% in sociodemographics, 0.3% in leisure activities, and 13–16% in the main dietary pattern. Being not accompanied by children (work in the city or abroad), participants living alone or cared for by neighbors contributed to some of the missing data. The mean substitution was used to handle missing values, and there was no significant statistic changing compared with analysis using original data on all substituted variables (22). Mean substitutions data can be seen in the Supplementary Tables. The leisure activity questionnaire was validated in several cities in Asia, including Bandung City in Indonesia; however, still there were no applicable activities such as visiting painting fairs, cinemas, music lounges, and libraries for the village setting. Though not much in quantity, they might also be sources of the difference in the number of recorded frequencies and examiners' errors.

Blood-based variables, such as plasma glucose and lipid profile, were obtained only in 57% due to refusal of participants/caregivers, including fear of blood taking, and chores like farming and selling in the morning. Binary logistic regression was applied to examine associations between potential risk factors and dementia. Univariable models were run for each potential risk factor separately, with adjustment for sex and education. The multivariable models included age, sex, education, and all risk factors in the univariable models. As data showed that the associations between lifestyle activities and dementia varied within age groups, the findings will be presented for the total sample and separately analyzed for the younger age group (60–74 years) and older age group (≥75 years). All the above data analyses were performed using the software IBM® SPSS 20.

## Results

### Demographic Characteristics of Participants

Six hundred eighty-six participants (296 male and 390 female) participated in this study. Two hundred participants (29.15%) fulfilled the criteria of dementia, which were found in 22.11% of elders in the suburban area and 39.42% of elders in the rural area. Participants with dementia were significantly older, had lower education, and were more likely to be female and single (*p* < 0.001) than those with no dementia. They had a lower socioeconomic status, such as lower occupational attainment and monthly income (*p* < 0.001). Moreover, they had worse health parameters such as more likely to be an active smoker (*p* < 0.023), with lower cognition, higher depression rate, more likely to be underweight (*p* < 0.001), and higher stroke event, but with a lower frequency of HDL < 40 mg/dl. Regarding lifestyle, this group was less active in all categories of leisure activities (*p* < 0.001), and they took less fruit (*p* < 0.001) (Table 1).

**Table 1:** Baseline characteristics of participants with and without dementia in Jatinangor cross-sectional study (*n* = 686).

	***n***	**No dementia(*n* = 486)**	**Dementia** **(*n* = 200)**	***p*-value**
Age (M ± SD)	686	68.29 ± 6.57	72.99 ± 7.21	<0.001
Sex (*n*, %)	686			<0.001
Male		232 (47.7)	64 (32.0)	
Female		254 (52.3)	136 (68.0)	
Education (M ± SD)	661	6.070 ± 3.08	3.605 ± 3.36	<0.001
Living area (*n*, %)	686			<0.001
Suburban		317 (65.2)	90 (45.0)	
Rural area		169 (34.8)	110 (55.0)	
Income (*n*, %)	598			<0.001
≥Monthly minimum wage		125 (25.7)	29 (14.5)	
< Monthly minimum wage		99 (20.4)	30 (15.0)	
No income		205 (42.2)	110 (55.0)	
Marital status (*n*, %)	629			<0.001
Still married		327 (67.3)	93 (46.5)	
Single (never married/divorced)		120 (24.7)	89 (44.5)	
Occupational class (*n*, %)	590			<0.001
Professional		134 (27.6)	28 (14.0)	
Not working		94 (19.3)	66 (33.0)	<0.001
Laborer		67 (13.8)	24 (12.0)	
Housewife		128 (26.3)	49 (24.5)	
Hypertension (*n*, %)	665	265 (54.5)	124 (62.0)	0.14
Stroke (*n*, %)	686	8 (1.6)	9 (4.5)	0.05
Diabetes (*n*, %)	391	34 (7.0)	16 (8.0)	0.74
Triglyceride > 200 mg/dl (*n*, %)	391	27 (5.6)	8 (4.0)	0.44
HDL <40 mg/dl (*n*, %)	391	53 (10.9)	12 (6.0)	0.04
BMI characteristics (*n*, %)	648			0.000
Normal		276 (56.8)	106 (53.0)	
Underweight		74 (15.2)	56 (28.0)	
Overweight		98 (20.2)	14 (7.0)	
Obese		20 (4.1)	4 (2.0)	
Smoking status (*n*, % active smoker)	506	169 (34.8)	52 (26.0)	0.02
Depression (*n*, %)	676	18 (3.7)	22 (11.0)	<0.001
Cognitive score AMT (M ± SD)	638	8.26 ± 1.34	4.53 ± 1.74	<0.001
Leisure activities (*n*, %)	684			
Intellectually less active		312 (64.2)	174 (87.0)	<0.001
Socially less active		228 (46.9)	131 (65.5)	<0.001
Recreationally less active		235 (48.4)	138 (69.0)	<0.001
Physically less active		411 (84.6)	188 (94.0)	<0.001
Less active (total leisure activity)				<0.001
**Dietary Intake (*****n*****, % non-frequent)**				
Carbohydrate intake	602	209 (43.0)	99 (49.5)	0.07
Protein intake	602	191 (39.3)	87 (43.5)	0.24
Vegetable intake	597	214 (44.0)	89 (44.5)	0.72
Fruit intake	579	302 (62.1)	143 (71.5)	0.001
Salted fish intake	523	109 (22.4)	42 (21.0)	0.75
Instant noodle intake	537	177 (36.4)	80 (40.0)	0.25
Tempe (fermented soybean) intake	530	177 (36.4)	65 (32.5)	0.50

*Non-parametric variables were compared using the Mann–Whitney test. Categorical variables are described as numbers and percentages, and groups were compared using the chi-squared test and Cramer's V test*.

*M, mean; n, number of participants with available data; SD, standard deviation; HDL, high-density lipoprotein; BMI, body mass index; AMT, Abbreviated Mental Test*.

In the total sample, univariable model showed statistical significance in risk factors of age, education level, living area, marital status, stroke, diabetes mellitus, underweight or overweight, depression, all categories of leisure activities, and non-frequent fruit intake (Table 2). In the multivariable model, the association remained significant in risk factors for age (>75 vs. 60–74 years: OR = 2.75, 95% CI = 1.70–4.4.6), marital status (single, never married/divorced vs. still married: OR = 1.72, 95% CI = 1.03–2.84), occupation (not working vs. professional: OR = 2.18, 95% CI = 1.04–4.61), BMI characteristic (overweight vs. normal: OR = 0.39: 95% CI = 0.18–0.82), intellectual activities (less active vs. active: OR = 2.85, 95% CI = 1.60–5.08), recreational activities (less active vs. active: OR = 2.19, 95% CI = 1.39–3.46), and fruit intake (non-frequent vs. frequent: OR = 2.02, 95% CI = 1.09–3.72) (Table 3).

**Table 2:** Univariable associations between potential risk factors and dementia in the total sample (*n* = 686) and stratified by age.

	**Total sample**		**60–74 years**		**≥75 years**	
	**OR**	95% CI^*^	**OR**	95% CI^*^	**OR**	95% CI^*^
Age						
60–74 years	1					
≥75 years	3.76	2.54–5.57				
Sex						
Male	1		1		1	
Female	1.52	0.93–2.50	1.34	0.86–2.08	5.04	2.48–10.25
Education						
≥7 years of education	1		1		1	
0–6 years of education	1.80	1.11–2.92	1.11	0.64–1.91	2.28	0.54–9.64
Living area						
Suburban	1		1		1	
Rural	2.26	1.58–3.24	2.22	1.42–3.46	2.10	1.00–4.37
Income						
≥Minimum wage	1		1		1	
< Minimum wage	1.24	0.65–2.36	2.13	1.07–4.23	1.39	0.39–4.93
No income	1.69	0.97–2.94	2.13	1.07–4.23	1.40	0.50–3.92
Marital status						
Still married	1		1		1	
Single (never married/divorced)	2.31	1.54–3.45	1.75	1.06–2.90	1.72	0.69–4.27
Occupational class						
Professional	1		1		1	
Not working	2.43	1.33–4.45	2.87	1.33–6.21	1.01	0.28–3.66
Laborer	1.97	0.97–4.02	2.91	1.20–7.06	0.63	0.15–2.60
Housewife	1.57	0.68–3.64	2.15	0.69–6.67	1.41	0.20–9.86
Hypertension	1.26	0.88–1.79	1.08	0.71–1.67	1.38	0.66–2.91
Stroke	3.77	1.20–11.84	3.07	0.80–11.74	8.18	0.71–93.37
Diabetes	0.95	2.71–7.86	1.16	0.50–2.80	0.54	0.14–2.01
Triglyceride > 200 mg/dl (*n*, %)	0.81	0.33–2.02	0.67	0.25–1.86	1.93	0.10–35.88
HDL <40 mg/dl (*n*, %)	0.60	0.29–1.23	0.78	0.36–1.70	0.44	0.07–2.78
BMI characteristics						
Normal	1		1		1	
Underweight	1.87	1.21–2.86	2.07	1.21–3.55	1.36	0.59–3.14
Overweight	0.33	0.17–0.63	0.46	0.23–0.93	0.20	0.03–1.11
Obese	0.46	0.15–1.40	0.54	0.15–1.94	1.04	0.05–20.38
Smoking status	1.30	0.66–2.54	1.01	0.46–2.20	5.17	0.62–43.86
Depression	2.53	1.25–5.11	3.75	1.63–8.66	1.09	0.33–3.61
Leisure activities						
Intellectually less active	3.50	2.61–5.68	2.96	1.70–5.16	3.09	1.01–9.46
Socially less active	2.04	1.43–2.91	1.79	1.16–2.77	1.52	0.71–3.28
Recreationally less active	2.99	2.06–4.34	2.43	1.56–3.79	2.38	1.05–5.41
Physically less active	3.26	1.63–6.49	2.40	1.11–5.18	3.70	0.70–19.57
Less active (total leisure activity)	3.16	2.19–4.58	2.67	1.71–4.16	2.75	1.26–6.01
Dietary intake						
Non-frequent Carbohydrate intake	1.32	0.92–1.90	1.55	0.98–2.45	0.81	0.38–1.71
Non-frequent protein intake	1.16	0.81–1.67	1.24	0.79–1.94	0.69	0.32–1.44
Non-frequent fruit intake	2.17	1.31–3.59	2.02	1.09–3.74	2.55	0.91–7.21
Non-frequent vegetable intake	1.02	0.70–1.47	0.99	0.62–1.57	1.03	0.50–2.16

*OR, odds ratio; 95% CI, 95% confidence interval; HDL, high-density lipoprotein; BMI, body mass index*.

* *Associations were adjusted for sex and education*.

**Table 3:** Multivariable models for the total sample (*n* = 686) and stratified by age.

	**Total sample**		**60–74 years**		**≥75 years**	
	**OR**	95% CI*	**OR**	95% CI*	**OR**	95% CI*
Age						
60–74 years	1					
≥75 years	2.75	1.70–4.46				
Sex						
Male	1		1		1	
Female	1.45	0.84–2.51	1.14	0.61–2.14	2.33	0.73–7.42
Education						
≥7 years of education	1		1		1	
0–6 years of education	0.52	0.27–1.00	0.45	0.22–0.91	1.81	0.26–12.72
Living area						
Suburban	1		1		1	
Rural	1.89	1.20–2.97	1.65	0.97–2.83	2.52	1.032–6.16
Income						
≥Minimum wage	1		1		1	
< Minimum wage	0.96	0.46–2.01	0.93	0.38–2.28	0.98	0.22–4.36
No income	1.36	0.54–3.39	1.38	0.44–4.27	1.28	0.21–7.69
Marital status						
Still married	1		1		1	
Single (never married/divorced)	1.71	1.03–2.84	1.53	0.85–2.276	2.80	0.89–8.877
Occupational class						
Professional	1		1		1	
Not working	2.18	1.03–4.60	2.98	1.21–7.31	1.02	0.22–4.65
Laborer	2.19	0.94–5.10	3.65	1.29–10.30	0.23	0.03–1.93
Housewife	1.89	0.64–5.56	2.43	0.68–8.71	0.87	0.06–12.43
Hypertension	1.12	0.73–1.73	1.10	0.66–1.81	1.29	0.52–3.20
Stroke	3.34	0.63–17.65	1.88	0.29–12.15		
Diabetes	1.24	0.53–2.86	1.61	0.58–4.49	0.67	0.15–3.05
Triglyceride > 200 mg/dl	1.37	0.48–3.93	1.09	0.36–3.29		
HDL <40 mg/dl	0.50	0.21–1.18	0.64	0.26–1.60	0.30	0.02–4.15
BMI characteristics						
Normal	1		1		1	
Underweight	1.30	0.78–2.19	1.49	0.80–2.80	1.25	0.48–3.26
Overweight	0.39	0.18–0.82	0.40	0.17–0.91	0.38	0.06–2.43
Obese	0.87	0.26–2.93	0.80	0.21–3.09	0.49	0.02–11.44
Smoking status	1.39	0.60–3.19	1.14	0.45–2.88	2.04	0.20–20.16
Depression	1.52	0.64–3.57	2.54	0.90–7.15	0.69	0.16–2.90
Leisure activities						
Intellectually less active	2.85	1.60–5.08	2.66	1.42–4.10	3.73	0.81–17.03
Socially less active	1.38	0.89–2.13	1.53	0.92–2.53	1.06	0.41–2.74
Recreationally less active	2.19	1.39–3.46	2.11	1.26–3.56	2.44	0.87–6.79
Physically less active	2.05	0.99–4.25	1.87	0.83–4.20	2.57	0.45–14.65
Less active (total leisure activity)	2.21	1.42–3.44	2.20	1.32–3.66	2.21	0.84–5.80
Dietary intake						
Non-frequent carbohydrate intake	1.13	0.74–1.74	1.30	0.78–2.15	1.03	0.43–2.45
Non-frequent protein intake	0.80	0.52–1.25	0.94	0.56–1.57	0.56	0.23–1.39
Non-frequent fruit intake	2.02	1.10–3.72	1.74	0.88–3.47	4.62	1.18–18.02
Non-frequent vegetable intake	0.96	0.62–1.50	0.62	0.57–1.62	1.03	0.43–2.45

*Multivariable models include age, sex, and education, plus all risk factors with ORs < 0.75 or >1.40 in the univariable models (Table 2)*.

*OR, odds ratio; 95% CI, 95% confidence interval; HDL, high-density lipoprotein; BMI, body mass index*.

In the younger age group (60–74 years), statistically significant univariable association with dementia was found for risk factors living area, marital status, occupation attainment, underweight/overweight, depression, all categories of leisure activities, and fruit intake (Table 2). In the multivariable model, the association with dementia remained significant in risk factors for occupational attainment (not working vs. professional: OR = 2.98, 95% CI = 1.21–7.31; labor vs. professional: OR = 3.65, 95% CI = 1.29–10.30), BMI (overweight vs. Normal: OR = 0.39, 95% CI = 0.17–0.90), intellectual activities (less active vs. active: OR = 2.67, 95% CI = 1.42–4.99), recreational activities (less active vs. active: OR = 2.12, 95 CI = 1.267–3.57), and total categories of leisure activities (less active vs. active: OR = 2.21, 95% CI = 1.42–3.44) (Table 3).

In the older age group (>75 years), a univariable association with dementia was found in risk factors sex, living area, and intellectual, recreational, and total categories of leisure activities (Table 2). In the multivariable model, association with dementia was found in living area (suburban vs. rural: OR = 2.52, 95% CI = 1.04–6.16) and fruit intake (non-frequent vs. frequent: OR = 4.62, 95% CI = 1.18–18.02) (Table 3).

## Discussion

### Prevalence of Dementia

The prevalence of dementia among Asian countries in 2012 varied from 0.03 to 33.2% (23). A study from Singapore showed that the prevalence was significantly higher among Indians (1.9%) compared with Malays (1.6%) and Chinese (1.2%) (24). A recent meta-analysis on the prevalence of dementia in six developing countries (Brazil, India, Jamaica, Kenya, Mexico, and South Africa) showed that the prevalence was 2–9% (25). A higher prevalence of dementia was reported recently from our province of Yogyakarta, 20.1%, varying from 13.9% in the city to 16.8–29.4% in other regencies (26). In the present study, the prevalence of dementia was 29.15, 22.11% of elders in the suburban area, and 39.42% of elders in the rural area. This result was consistent with the abovementioned report from Yogyakarta (26). Differences in dementia diagnosis methods and demographic, socioeconomic, and risk factors may cause variation in the prevalence of dementia. Both studies from our country used local validated cognitive tests (MMSE and AMT), with AD8 as informant report of cognitive decline, and utilized ADL/IADL score for detecting functional decline.

Moreover, many participants living in rural areas with low economic status might contribute to the higher prevalence in our country than in other countries mentioned above. The present study showed that, compared with those residing in suburban, participants living in rural areas were significantly older and had lower education levels and lower occupational attainment (more likely to be not working and less likely to be professionals). These participants were less active in leisure activities, including physical training, social involvement, recreation and relaxation, and cognitive stimulation than were their counterpart elders in suburban. Association between work, social engagement, and mental stimulation has been reported in a previous study (27). Furthermore, the participants took fewer fruits, which are rich in antioxidants and good for cognition (6). These findings further enrich our insight that aside from education level and occupation attainment, as reported in a previous study (28), less engagement in leisure activities and poor dietary patterns may also associate with a higher risk of dementia.

### Risk Factors Associated With Dementia

In the present study, the risk factors associated with dementia included demographic, socioeconomic, and health factors and lifestyle, including leisure activities and dietary patterns. We found differences in the numbers and types of risk factors associated with dementia between the younger age group (60–74 years) and the older age group (>75 years). The younger group had more risk factors, including demographic, socioeconomic (occupation attainment), and health factors (BMI) and lifestyle, such as intellectual and recreational activities. Meanwhile, living in rural areas and lifestyles such as less fruit intake were the only risk factors in the older group.

### Age-Dependent Risk Factors for Cognitive Decline/Dementia

Most cardiovascular factors will lose their impact on cognitive decline at an older age than at a younger age, as reported in previous studies (29, 30). Midlife hypertension is a known risk factor for developing dementia and Alzheimer's disease in late life until 74 years (31, 32). However, it is protective against dementia at ages over 85 years (33, 34). It is noteworthy that low blood pressure can be a consequence of neurodegenerative disease; therefore, low blood pressure may be an early sign of dementia onset (34, 35). High cholesterol in late life can indicate a better nutritional status and better overall health, and therefore, it was associated with less cognitive decline (36, 37). Several possible mechanisms may explain the abovementioned phenomena (38). Risk factors may occur across the life span, and they take time to affect health in general and the brain in particular. Midlife may be the most critical period for the accumulation of these risk factors. Other chronic conditions, such as lifestyle, may occur during old age and cause “weakening” of the measured associations between vascular risk factors and dementia (39).

Moreover, individuals in old age often have dementia with mixed pathology, which is challenging to identify since certain risk factors may be relevant to specific pathology (40). Mortality bias should also be put into consideration. Old age dementia individuals with vascular risk factors may have a higher mortality rate than those younger, either with or without dementia. A recent report from a large cohort study from Europe has demonstrated the independent association of poor cognitive performance and higher mortality. The mortality was significantly higher in those with old age and comorbidities, such as heart attack, stroke, and diabetes, and lower socioeconomic class (41). Therefore, fewer risk factors will be found in old age compared with younger age groups.

### Lifestyle Factors

#### Leisure Activities

Leisure activity can be defined as the voluntary use of free time for activities outside the daily routine. The protective effect of mental activity on cognitive decline has been consistently reported in observational and interventional studies (21). In the present study, we found a significant association between all categories of leisure activities in the younger age group; meanwhile, only cognitive and recreational activities in the older age group were associated with dementia risk after controlling sex and education. However, as we controlled all health factors, only the cognitive and recreational activities remained related to the risk of dementia in the younger age group, but not in the older age group. Engagement in cognitive activities (such as book reading, playing board games, writing, and playing a musical instrument) and recreational activities (such as listening to music and radio, and shopping) seemed to protect the brain from dementia in this study population. A previous longitudinal study demonstrated that all three activities engaged, namely, cognitive, physical, and social, contribute equally to decreasing dementia risk (42). The exact protective mechanism of leisure activities is unknown. Hypothetically, leisure activities improve cognitive reserve, allowing more efficient, adaptive, and plastic neuronal processing to better cope with progressing dementia pathology (43). Physical movement and social activities can also promote cerebral blood flow and psychological well-being effects and reduce the inflammation process in the brain (44, 45).

#### Dietary Pattern

In a recently published meta-analysis study, the risk of cognitive impairment and dementia was reduced by 20% for a higher consumption of fruit and vegetables (6). In that study, fruit consumption or in combination with vegetable, but not vegetable consumption, was associated with the protection of cognitive impairment. This protective effect was significant only in participants age over 65 years and not in those younger (6). This age-specific effect of fruit take was similar to our findings, which showed that less fruit take was associated with a higher risk of dementia in older age group (≥75 years), but not in the younger age group (60–74 years). Both fruits and vegetables are rich in antioxidants such as carotenoids, α- and γ-tocopherol, and folate, which can protect the brain from free radical scavengers, hyper-homocysteinemia, and cognitive impairment (46).

None of the participants consumed alcohol in the present study, so we could not evaluate the effect of alcohol drinking on cognitive impairment. We did not find any association between dementia and the consumption of our traditional food tofu and tempeh, often consumed with salted fish or instant noodles, as well as the rarely consumed fish, in both age groups, as was reported by previous studies (47–50).

### Health Factors

In the present study, stroke was more prevalent in the dementia group than those without dementia (*p* < 0.05). Stroke tripled the risk of dementia (OR = 3.77, 95% CI = 1.2–11.86) in the univariable model after controlling for sex and education. Similar association studies also reported from other LMICs (51) and HICs (52). A recent study showed the age-specific effect of stroke on dementia, particularly in the younger age group (38). Stroke is the leading health problem in Indonesia since it occupied the first rank of cause of death in our national basic health research (RISKESDAS) in 2013 and 2018, which is 7 and 10.9%, respectively (53, 54). The small cases of stroke detected in the present study could be due to the under-recognition by participants and caregivers. The subtle motor symptoms of cerebral small vessel disease, such as lacunar infarction, were challenging to detect (55). Current evidence on post-stroke dementia showed that aside from stroke severity, the cognitive impairment after stroke also depends on the interaction between the inflammatory process after stroke and the existence of neurodegenerative pathologies, especially beta-amyloid (52, 56).

BMI analysis in the univariable model showed that, in the younger age group, underweight was associated with an increased risk of dementia (OR = 2.07, 95% CI = 1.21–3.56), and overweight was associated with lower risk dementia (OR = 0.46, 95% CI = 0.23–0.94). This finding should be interpreted carefully since it may be confounded by a long period (two to three decades) of preclinical dementia bodyweight loss. Therefore, the association between BMI and dementia is likely attributable to two different processes, a harmful effect of higher BMI, observable in the extended follow-up study, and a reverse-causation impact, which makes a higher BMI appear protective when the follow-up period is short (57).

In the present study, we did not find any significant relationship between hypertension, diabetes, and dyslipidemias with dementia. It did not mean that these risk factors did not play a role in the decline of cognitive function. As discussed before, the risk factors occur across the life span (most critically, midlife), accumulating the impact on the brain. Chronic conditions, such as leisure activities, stroke, BMI, and nutrition, might have overlapped (in the same group) and cumulatively “weakened” the measured associations between these vascular risk factors and dementia (39). Also, the non-linear association is challenging to be investigated in our cross-sectional study design. Moreover, the present study is underpowered with a limited sample size to examine these associations, even after doing age group analysis. A recent systematic review demonstrated that higher blood pressure was associated with a higher risk of cognitive decline in people without dementia and stroke (58).

Furthermore, the increase of 24-h ambulatory blood pressure variability was associated with lower cognitive function in elderly hypertension with well-controlled blood pressure (59). Higher daytime systolic blood pressure was associated with a 3.73 risk of dementia and a 10.54 risk of MRI finding of subcortical vascular dementia (60). All these data highlight the need for the early management of these risk factors, particularly the blood pressure, even in the absence of clinical hypertension to prevent the risk of cognitive decline typically associated with aging.

### Implication of the Study

The present study has demonstrated that both lifestyle and risk factors were associated with the risk of dementia in the district of Jatinangor, Sumedang Regency, in Indonesia. Findings that less engagement in leisure activities and poor dietary patterns are associated with a higher risk of dementia may open up new intervention opportunities for the elders and encourage primary prevention of dementia that started from a younger age. The fact that stroke associates reciprocally with dementia shed the hope that intervention of risk factors after stroke (secondary stroke prevention) and management of risk factors since a younger age (primary stroke prevention) may also reduce the prevalence of dementia in the future. The significance of multiple risk factors in our multivariate model suggests the additive effects on dementia risk. They imply the importance of multidomain intervention targeting all risk factors, alongside the improvement of socioeconomic status, as an integrated action in public policy on dementia prevention programs. We recommend large-scale research to obtain nationwide dementia prevalence and associated risk factors to design an effective and efficient national dementia prevention strategy in the future.

### Strengths and Limitation of This Study

Several limitations are worthy to be mentioned in the present study. The nature of a cross-sectional study design would not allow us to determine the causalities between variables firmly. Our finding may be underpowered, reflected in the wide confidence interval and the insignificancy of statistical analyses, especially in variables based on blood tests. Participants of the present study were recruited from suburban and rural areas; therefore, the current findings may be more relevant for the lower socioeconomic status population. Food registry and leisure activities were based on the recall of memory. A self-report by participants with cognitive decline may confound the associations. However, most of the caregivers were present and confirmed answers given by participants. This study's added value included multiple potential risk factors, including socioeconomic, health, and lifestyle factors with broad spectrums of leisure activities and dietary patterns, which might not be explored together before. At the same time, we may have missed other nutritional components, nutrients, and cognitive intervention.

## Conclusion

The prevalence of dementia in the present study is high. The risk factors associated with dementia are age-dependent. The younger group (60–74 years) has more risk factors, including lower education, lower occupational attainment, and less intellectual and recreational activities. The risk factors for the older age group (>75 years) are living in a rural area and less fruit intake. Identifying these modifiable lifestyle risk factors is crucial for designing effective interventions and determining public health policies for future dementia prevention in Indonesia.

## Data Availability Statement

The raw data supporting the conclusions of this article will be made available by the authors, without undue reservation.

## Ethics Statement

The Medical Ethics Committee of Universitas Padjadjaran Bandung, Indonesia approved this study design and protocol. The patients/participants provided their written informed consent to participate in this study. Written informed consent was obtained from the individual(s) for the publication of any potentially identifiable images or data included in this article.

## Author Contributions

PO, CC, NS, YS, and YD were responsible for the study design. FA and NP were responsible for the data analyses. PO was responsible for drafting the manuscript. All authors contributed to interpreting the findings, provided critical feedback on drafts of the manuscript, and approved the final manuscript.

## Conflict of Interest

The authors declare that the research was conducted in the absence of any commercial or financial relationships that could be construed as a potential conflict of interest.

## Publisher's Note

All claims expressed in this article are solely those of the authors and do not necessarily represent those of their affiliated organizations, or those of the publisher, the editors and the reviewers. Any product that may be evaluated in this article, or claim that may be made by its manufacturer, is not guaranteed or endorsed by the publisher.
