# Supplementary material for: Dementia Prevalence, Comorbidities, and Lifestyle Among Jatinangor Elders
Source: Front Neurol. 2021 Jul 23;12:643480. doi: 10.3389/fneur.2021.643480 (PMC8345013; doi:10.3389/fneur.2021.643480)
Supplement: Supplementary file 2 [file Table_1.docx]

| **Leisure Activity** | **n** | **Total**  **(n=686)** | **No Dementia**  **(n=486)** | **Incident Dementia**  **(n=200)** |
| --- | --- | --- | --- | --- |
| **Intellectual Activity (n, %)** | 684 |  |  |  |
| 1. Reading books, newspapers, or magazines |  | 318 (46.4) | 273 (56.2) | 45 (22.5) |
| 1. Using computer or surfing the internet |  | 9 (1.3) | 7 (1.4) | 2 (1.0) |
| 1. Playing boar games, cards, chess, and domino |  | 23 (3.4) | 19 (3.9) | 4 (2.0) |
| 1. Investment or following the stock market |  | 31 (4.5) | 25 (5.1) | 6 (3.0) |
| 1. Participating in forums or discussions |  | 215 (31.3) | 176 (36.2) | 39 (19.0) |
| 1. Writing, calligraphy, and painting |  | 85 (12.4) | 70 (14.4) | 15 (7.5) |
| 1. Playing a music instrument |  | 5 (0.7) | 3 (0.6) | 2 (1.0) |
| 1. Other intellectual activities |  | 13 (1.9) | 12 (2.5) | 1 (0.5) |
| Total of Intellectual Activity (M ± SD) |  | 1.02 ± 0.10 | 1.20 ± 0.99 | 0.57 ± 0.87 |
| **Social Activity (n, %)** | 684 |  |  |  |
| 1. Attending an interest class |  | 68 (9.9) | 58 (11.9) | 10 (5.0) |
| 1. Joining a social center (social community “pauyuban”, neighborhood regular meeting “arisan”) |  | 191 (27.8) | 150 (30.9) | 41 (20.5) |
| 1. Participating in volunteer work |  | 363 (52.9) | 284 (58.4) | 79 (39.5) |
| 1. Going to museums, exhibitions, theaters or concerts |  | 25 (3.6) | 23 (4.7) | 2 (1.0) |
| 1. Meeting relatives or friends |  | 334 (48.7) | 245 (50.4) | 89 (44.5) |
| 1. Drama or opera performance |  | 21 (3.1) | 14 (2.9) | 7 (3.5) |
| 1. Singing |  | 94 (13.7) | 75 (15.4) | 19 (9.5) |
| 1. Attending religious activities |  | 550 (80.2) | 402 (82.7) | 148 (74.0) |
| 1. Other social activities |  | 16 (2.3) | 13 (2.7) | 3 (1.5) |
| Total of Social Activity (M ± SD) |  | 2.43 ± 1.49 | 2.60 ± 1.42 | 2.01 ±1.29 |
| **Recreational Activity (n, %)** | 684 |  |  |  |
| 1. Watching television and listening to the radio |  | 588 (85.7) | 440 (90.5) | 148 (74.0) |
| 1. Listening to music |  | 380 (55.4) | 297 (61.1) | 83 (41.5) |
| 1. Shopping |  | 401 (58.5) | 308 (63.4) | 93 (46.5) |
| 1. Cooking for pleasure |  | 324 (47.2) | 236 (48.6) | 88 (44.0) |
| 1. Fishing |  | 35 (5.1) | 33 (6.8) | 2 (1.0) |
| 1. Keeping plants or pets |  | 312 (45.5) | 244 (50.2) | 68 (34.0) |
| 1. Facial or massage |  | 166 (24.2) | 128 (26.3) | 38 (19.0) |
| 1. Other recreational activities |  | 10 (1.5) | 9 (1.9) | 1 (0.5) |
| Total of Recreational Activity (M ± SD) |  | 3.23 ±1.61 | 3.49 ± 1.58 | 2.63 ± 1.51 |
| **Physical** **Activity (n, %)** | 684 |  |  |  |
| 1. Mind-body exercise |  | 4 (0.6) | 4 (0.8) | 0(0.0) |
| Tai Chi, Qi Gong, Yoga, other mind-body exercise(s) |  |  |  |  |
| 1. Light aerobic exercise |  | 67 (9.8) | 59 (12.1) | 8 (4.0) |
| Brain exercise, osteoporosis exercise, diabetic exercise, elderly exercise. |  |  |  |  |
| 1. Strenuous aerobic exercise |  | 73 (10.6) | 62 (12.8) | 11 (5.5) |
| Martial arts, jogging or running, stair climbing, swimming, hiking or excursions, bicycling or using exercise machines, Playing ballgames or racquet sports, calisthenics, dancing |  |  |  |  |
| 1. Stretching and toning exercise |  | 307 (44.8) | 240 (49.4) | 67 (33.5) |
| Slow walking, Pebble trail walking, General Stretching, Toning exercise |  |  |  |  |
| 1. Other Physical activities |  | 24 (3.5) | 18 (3.7) | 6 (3.0) |
| Total of Physical Activity (M ± SD) |  | 0.70 ± 0.74 | 0.79 ± 0.76 | 0.46 ± 0.65 |
| **Total of Leisure Activity** |  | 7.38 ± 3.50 | 8.08 ± 3.43 | 5.68 ± 2.07 |

**Table 4**. Leisure Activity Pattern

*M, mean; n, number of participants with available data; SD, standard deviation*

**Table 5**. Leisure Activity stratified by age

| **Leisure Activity** | **n** | **Total**  **(n=686)** | **60-74 years**  **(n=515)** | **≥ 75 years**  **(n=161)** |
| --- | --- | --- | --- | --- |
| **Intellectual Activity (n, %)** | 674 |  |  |  |
| 1. Reading books, newspapers, or magazines |  | 318 (46.4) | 261 (50.7) | 51 (31.7) |
| 1. Using computer or surfing the internet |  | 9 (1.3) | 7 (1.4) | 2 (1.2) |
| 1. Playing boar games, cards, chess, and domino |  | 23 (3.4) | 19 (3.7) | 2 (1.9) |
| 1. Investment or following the stock market |  | 31 (4.5) | 25 (4.9) | 6 (3.7) |
| 1. Participating in forums or discussions |  | 215 (31.3) | 174 (33.8) | 37 (23.0) |
| 1. Writing, calligraphy, and painting |  | 85 (12.4) | 67 (13.0) | 16 (9.9) |
| 1. Playing a music instrument |  | 5 (0.7) | 5 (1.0) | 0 (0.0) |
| 1. Other intellectual activities |  | 13 (1.9) | 10 (1.9) | 3 (1.9) |
| Total of Intellectual Activities (M ± SD) |  | 1.02 ± 0.10 | 1.10 ± 1.00 | 0.74 ± 0.90 |
| **Social Activity (n, %)** | 674 |  |  |  |
| 1. Attending an interest class |  | 68 (9.9) | 58 (11.3) | 10 (6.2) |
| 1. Joining a social center (social community “paguyuban”, neighborhood regular meeting “arisan”) |  | 191 (27.8) | 149 (28.9) | 38 (23.6) |
| 1. Participating in volunteer work |  | 363 (52.9) | 294 (57.1) | 65 (40.4) |
| 1. Going to museums, exhibitions, theaters or concerts |  | 25 (3.6) | 21 (4.1) | 2 (2.5) |
| 1. Meeting relatives or friends |  | 334 (48.7) | 266 (51.7) | 61 (37.9) |
| 1. Drama or opera performance |  | 21 (3.1) | 18 (3.5) | 3 (1.9) |
| 1. Singing |  | 94 (13.7) | 82 (15.9) | 11 (6.8) |
| 1. Attending religious activities |  | 550 (80.2) | 425 (82.5) | 117 (72.7) |
| 1. Other social activities |  | 16 (2.3) | 13 (2.5) | 3 (1.9) |
| Total of Social Activities (M ± SD) |  | 2.43 ± 1.49 | 2.57 ± 1.40 | 1.96 ± 1.34 |
| **Recreational Activity (n, %)** | 674 |  |  |  |
| 1. Watching television and listening to the radio |  | 588 (85.7) | 465 (90.3) | 114 (70.8) |
| 1. Listening to music |  | 380 (55.4) | 305 (59.2) | 70 (43.5) |
| 1. Shopping |  | 401 (58.5) | 326 (63.3) | 70 (43.5) |
| 1. Cooking for pleasure |  | 324 (47.2) | 263 (51.1) | 56 (34.8) |
| 1. Fishing |  | 35 (5.1) | 32 (6.2) | 3 (1.9) |
| 1. Keeping plants or pets |  | 312 (45.5) | 260 (50.5) | 49 (30.4) |
| 1. Facial or massage |  | 166 (24.2) | 137 (26.6) | 29 18.0) |
| 1. Other recreational activities |  | 10 (1.5) | 8 (1.6) | 1 (0.6) |
| Total of Recreational Activities (M ± SD) |  | 3.23 ±1.61 | 3.48 ± 1.54 | 2.46 ± 1.56 |
| **Physical** **Activity (n, %)** | 674 |  |  |  |
| 1. Mind-body exercise |  | 4 (0.6) | 4 (0.8) | 0 (0.0) |
| Tai Chi, Qi Gong, Yoga, other mind-body exercise(s) |  |  |  |  |
| 1. Light aerobic exercise |  | 67 (9.8) | 56 (10.9) | 8 (5.0) |
| Brain exercise, osteoporosis exercise, diabetic exercise, elderly exercise. |  |  |  |  |
| 1. Strenuous aerobic exercise |  | 73 (10.6) | 63 (12.2) | 10 (6.2) |
| Martial arts, jogging or running, stair climbing, swimming, hiking or excursions, bicycling or using exercise machines, Playing ballgames or racquet sports, calisthenics, dancing |  |  |  |  |
| 1. Stretching and toning exercise |  | 307 (44.8) | 242 (47.0) | 58 (36.0) |
| Slow walking, Pebble trail walking, General Stretching, Toning exercise |  |  |  |  |
| 1. Other Physical activities |  | 24 (3.5) | 20 (3.9) | 4 (2.5) |
| Total of Physical Activities (M ± SD) |  | 0.70 ± 0.74 | 0.74 ± 0.75 | 0.50 ± 0.67 |
| **Total of Leisure Activities** |  | 7.38 ± 3.50 | 7.91 ± 3.42 | 5.67 ± 3.20 |

*M, mean; n, number of participants with available data; SD, standard deviation*

**Table 6**. Dietary Intake Pattern

| **Dietary Intake** | **n** | **Total**  **(n=686)** | **n** | **No Dementia**  **(n=486)** | **n** | **Incident Dementia**  **(n=200)** |
| --- | --- | --- | --- | --- | --- | --- |
| **Carbohydrate intake (n, %)** | 602 |  | 429 |  | 173 |  |
| 1 time/day |  | 17 (2.5) |  | 8 (1.6) |  | 9 (4.5) |
| 2 times/day |  | 291 (42.4) |  | 201 (41.4) |  | 90 (45.0) |
| 3 times/day |  | 294 (42.9) |  | 220 (45.3) |  | 74 (37.0) |
| **Protein intake (n, %)** | 602 |  | 428 |  | 174 |  |
| Never |  | 7 (1.0) |  | 6 (1.2) |  | 1 (0.5) |
| Sometimes |  | 168 (24.5) |  | 118 (24.3) |  | 50 (25.0) |
| 1 time/week |  | 35 (5.1) |  | 23 (4.7) |  | 12 (6.0) |
| 3-4 times/week |  | 68 (9.9) |  | 44 (9.1) |  | 24 (12.0) |
| 1 time/day |  | 107 (15.6) |  | 74 (15.2) |  | 33 (16.5) |
| 2 times/day |  | 129 (18.8) |  | 95 (19.5) |  | 34 (17.0) |
| 3 times/day |  | 88 (12.8) |  | 68 (14.0) |  | 20 (10.0) |
| **Fruit Intake (n, %)** | 579 |  | 413 |  | 166 |  |
| Never |  | 7 (1.0) |  | 4 (0.8) |  | 3 (1.5) |
| Sometimes |  | 355 (51.7) |  | 238 (49.0) |  | 117 (58.5) |
| 1 time/week |  | 45 (6.6) |  | 31 (6.4) |  | 14 (7.0) |
| 3-4 times/week |  | 38 (5.5) |  | 29 (6.0) |  | 9 (4.5) |
| 1 time/day |  | 94 (13.7) |  | 76 (15.6) |  | 18 (9.0) |
| 2 times/day |  | 22 (3.2) |  | 19 (3.9) |  | 3 (1.5) |
| 3 times/day |  | 18 (2.6) |  | 16 (3.3) |  | 2 (1.0) |
| **Vegetable intake (n, %)** | 597 |  | 426 |  | 171 |  |
| Never |  | 1 (0.1) |  | 0 (0.0) |  | 1 (0.5) |
| Sometimes |  | 198 (28.9) |  | 146 (30.0) |  | 52 (26.0) |
| 1 time/week |  | 45 (6.6) |  | 26 (5.3) |  | 19 (9.5) |
| 3-4 times/week |  | 59 (8.6) |  | 42 (8.6) |  | 17 (8.5) |
| 1 time/day |  | 121 (17.6) |  | 84 (17.3) |  | 37 (18.5) |
| 2 times/day |  | 113 (16.5) |  | 82 (16.9) |  | 31 (15.5) |
| 3 times/day |  | 60 (8.7) |  | 46 (9.5) |  | 14 (7.0) |
| **Salted Fish intake (n, %)** | 523 |  | 371 |  | 152 |  |
| Sometimes |  | 151 (22.0) |  | 109 (22.4) |  | 42 (21.0) |
| Few times/month |  | 37 (5.4) |  | 23 (4.7) |  | 14 (7.0) |
| Few times/week |  | 151 (22.0) |  | 105 (21.6) |  | 46 (23.0) |
| 1-2 times/day |  | 122 (17.8) |  | 90 (18.5) |  | 32 (16.0) |
| 3 times/day |  | 62 (9.0) |  | 44 (9.1) |  | 18 (9.0) |
| **Instant Noodle intake (n, %)** | 537 |  | 383 |  | 154 |  |
| Sometimes |  | 257 (37.5) |  | 177 (36.4) |  | 80 (40.0) |
| Few times/month |  | 111 (16.2) |  | 75 (15.4) |  | 36 (18.0) |
| Few times/week |  | 153 (22.3) |  | 120 (24.7) |  | 33 (16.5) |
| 1-2 times/day |  | 9 (1.3) |  | 5 (1.0) |  | 4 (2.0) |
| 3 times/day |  | 7 (1.0) |  | 6 (1.2) |  | 1 (0.5) |
| **Tempe (Fermented soy bean) intake (n, %)** | 530 |  | 379 |  | 151 |  |
| Sometimes |  | 52 (7.6) |  | 40 (8.2) |  | 12 (6.0) |
| Few times/month |  | 35 (5.1) |  | 30 (6.2) |  | 5 (2.5) |
| Few times/week |  | 155 (22.6) |  | 107 (22.0) |  | 48 (24.0) |
| 1-2 times/day |  | 207 (30.2) |  | 149 (30.7) |  | 58 (29.0) |
| 3 times/day |  | 81 (11.8) |  | 53 (10.9) |  | 28 (14.0) |

*n, number of participants with available data*

**Table 7**. Dietary Intake Pattern Stratified by Age

| **Dietary Intake** | **n** | **Total**  **(n=686)** | **n** | **60-74 years**  **(n=515)** | **n** | **≥ 75 years**  **(n=161)** |
| --- | --- | --- | --- | --- | --- | --- |
| **Carbohydrate intake (n, %)** | 599 |  | 461 |  | 138 |  |
| 1 time/day |  | 17 (2.5) |  | 9 (1.7) |  | 8 (5.0) |
| 2 times/day |  | 289 (42.8) |  | 221 (42.9) |  | 68 (42.2) |
| 3 times/day |  | 293 (43.3) |  | 231 (44.9) |  | 62 (38.5) |
| **Protein intake (n, %)** | 599 |  | 460 |  | 139 |  |
| Never |  | 7 (1.0) |  | 5 (1.0) |  | 2 (1.2) |
| Sometimes |  | 166 (24.6) |  | 123 (23.9) |  | 43 (26.7) |
| 1 time/week |  | 35 (5.2) |  | 24 (4.7) |  | 11 (6.8) |
| 3-4 times/week |  | 68 (10.1) |  | 48 (9.3) |  | 20 (12.4) |
| 1 time/day |  | 107 (15.8) |  | 85 (16.5) |  | 22 (13.7) |
| 2 times/day |  | 128 (18.9) |  | 101 (19.6) |  | 27 (16.8) |
| 3 times/day |  | 88 (13.0) |  | 74 (14.4) |  | 14 (8.7) |
| **Fruit Intake (n, %)** | 594 |  | 442 |  | 134 |  |
| Never |  | 7 (1.0) |  | 5 (1.0) |  | 2 (1.2) |
| Sometimes |  | 354 (52.4) |  | 263 (51.1) |  | 91 (56.5) |
| 1 time/week |  | 44 (6.5) |  | 33 (6.4) |  | 11 (6.8) |
| 3-4 times/week |  | 38 (5.6) |  | 29 (5.6) |  | 9 (5.6) |
| 1 time/day |  | 93 (13.8) |  | 78 (15.1) |  | 15 (9.3) |
| 2 times/day |  | 22 (3.3) |  | 17 (3.3) |  | 5 (3.1) |
| 3 times/day |  | 18 (2.7) |  | 17 (3.3) |  | 1 (0.6) |
| **Vegetable intake (n, %)** | 576 |  | 455 |  | 139 |  |
| Never |  | 1 (0.1) |  | 0 (0.0) |  | 1 (0.6) |
| Sometimes |  | 197 (29.1) |  | 155 (30.1) |  | 42 (26.1) |
| 1 time/week |  | 45 (6.7) |  | 26 (5.0) |  | 19 (11.8) |
| 3-4 times/week |  | 59 (8.7) |  | 44 (8.5) |  | 15 (9.3) |
| 1 time/day |  | 120 (17.8) |  | 101 (19.6) |  | 19 (11.8) |
| 2 times/day |  | 112 (16.6) |  | 77 (15.0) |  | 35 (21.7) |
| 3 times/day |  | 60 (8.9) |  | 52 (10.1) |  | 8 (5.0) |
| **Salted Fish intake (n, %)** | 534 |  | 400 |  | 120 |  |
| Sometimes |  | 149 (22.0) |  | 115 (22.3) |  | 34 (21.1) |
| Few times/month |  | 37 (5.5) |  | 26 (5.0) |  | 11 (6.8) |
| Few times/week |  | 150 (22.2) |  | 115 (22.3) |  | 35 (21.7) |
| 1-2 times/day |  | 122 (18.0) |  | 94 (18.3) |  | 28 (17.4) |
| 3 times/day |  | 62 (9.2) |  | 50 (9.7) |  | 12 (7.5) |
| **Instant Noodle intake (n, %)** | 520 |  | 411 |  | 123 |  |
| Sometimes |  | 255 (37.7) |  | 187 (36.3) |  | 68 (42.2) |
| Few times/month |  | 110 (16.3) |  | 83 (16.1) |  | 27 (16.8) |
| Few times/week |  | 153 (22.6) |  | 129 (25.0) |  | 24 (14.9) |
| 1-2 times/day |  | 9 (1.3) |  | 7 (1.4) |  | 2 (1.2) |
| 3 times/day |  | 7 (1.0) |  | 5 (1.0) |  | 2 (1.2) |
| **Tempe (Fermented soy bean) intake (n, %)** | 527 |  | 400 |  | 120 |  |
| Sometimes |  | 52 (7.7) |  | 115 (22.3) |  | 34 (21.1) |
| Few times/month |  | 35 (5.2) |  | 26 (5.0) |  | 11 (6.8) |
| Few times/week |  | 154 (22.8) |  | 115 (22.3) |  | 35 (21.7) |
| 1-2 times/day |  | 206 (30.5) |  | 94 (18.3) |  | 28 (17.4) |
| 3 times/day |  | 80 (11.8) |  | 50 (9.7) |  | 12 (7.5) |

*n, number of participants with available data*

|  | n | No Dementia  (n=486) | Dementia  (n=200) | p-value |
| --- | --- | --- | --- | --- |
| Age (M±SD) | 686 | 68.29 ± 6.57 | 72.99 ± 7.21 | < 0.001 |
| Sex (n, %) | 686 |  |  | < 0.001 |
| Male |  | 232 (47.7) | 64 (32.0) |  |
| Female |  | 254 (52.3) | 136 (68.0) |  |
| Education (M±SD) | 686 | 6.070 ± 3.08 | 3.605 ± 3.36 | < 0.001 |
| Living area (n, %) | 686 |  |  | < 0.001 |
| Suburban |  | 317 (65.2) | 90 (45.0) |  |
| Rural area |  | 169 (34.8) | 110 (55.0) |  |
| Income (n, %) | 598 |  |  | < 0.001 |
| ≥ monthly minimum wage |  | 125 (25.7) | 29 (14.5) |  |
| < monthly minimum wage |  | 99 (20.4) | 30 (15.0) |  |
| No Income |  | 205 (42.2) | 110 (55.0) |  |
| Marital Status (n, %) | 629 |  |  | < 0.001 |
| Still married |  | 327 (67.3) | 93 (46.5) |  |
| Single (never married/divorce) |  | 120 (24.7) | 89 (44.5) |  |
| Occupational class (n, %) | 590 |  |  | < 0.001 |
| Professional |  | 134 (27.6) | 28 (14.0) |  |
| Not working |  | 94 (19.3) | 66 (33.0) | < 0.001 |
| Laborer |  | 67 (13.8) | 24 (12.0) |  |
| Housewife |  | 128 (26.3) | 49 (24.5) |  |
| Hypertension (n, %) | 686 | 265 (54.5) | 124 (62.0) | 0.14 |
| Stroke (n, %) | 686 | 8 (1.6) | 9 (4.5) | 0.05 |
| Diabetes (n, %) | 686 | 34 (7.0) | 16 (8.0) | 0.74 |
| Triglyceride > 200mg/dl (n, %) | 686 | 27 (5.6) | 8 (4.0) | 0.44 |
| HDL < 40mg/dl (n, %) | 686 | 53 (10.9) | 12 (6.0) | 0.04 |
| BMI Characteristics (n, %) | 686 |  |  | 0.000 |
| Normal |  | 276 (56.8) | 106 (53.0) |  |
| Underweight |  | 74 (15.2) | 56 (28.0) |  |
| Overweight |  | 98 (20.2) | 14 (7.0) |  |
| Obese |  | 20 (4.1) | 4 (2.0) |  |
| Smoking status (n, % active smoker) | 506 | 169 (34.8) | 52 (26.0) | 0.02 |
| Depression (n, %) | 686 | 18 (3.7) | 22 (11.0) | < 0.001 |
| Cognitive Score AMT (M±SD) | 686 | 8.26 ± 1.34 | 4.53 ± 1.74 | < 0.001 |
| Leisure activities (n, %) | 686 |  |  |  |
| Intellectually less active |  | 312 (64.2) | 174 (87.0) | < 0.001 |
| Socially less active |  | 228 (46.9) | 131 (65.5) | < 0.001 |
| Recreationally less active |  | 235 (48.4) | 138 (69.0) | < 0.001 |
| Physically less active |  | 411 (84.6) | 188 (94.0) | < 0.001 |
| Less active (total leisure activity) |  |  |  | < 0.001 |
| Dietary Intake (n, % non-frequent) |  |  |  |  |
| Carbohydrate intake | 686 | 209 (43.0) | 99 (49.5) | 0.07 |
| Protein intake | 686 | 191 (39.3) | 87 (43.5) | 0.24 |
| Vegetable intake | 686 | 214 (44.0) | 89 (44.5) | 0.72 |
| Fruit intake | 686 | 302 (62.1) | 143 (71.5) | 0.001 |
| Salted Fish intake | 686 | 109 (22.4) | 42 (21.0) | 0.75 |
| Instant Noodle intake | 686 | 177 (36.4) | 80 (40.0) | 0.25 |
| Tempe (fermented soybean) intake | 686 | 177 (36.4) | 65 (32.5) | 0.50 |

**Table 8.** Baseline characteristics of participants with and without dementia in Jatinangor cross-sectional study (n=686) with missing data handled by mean substitution

*M, mean; n, number of participants with available data; SD, standard deviation Non-parametric variables were compared using the Mann-Whitney test. Categorical variables were described as numbers and percentages, and groups were compared using the Chi-squared test and Cramer’s V test*

|  | Total Sample | | 60-74 years | | ≥75 years | |
| --- | --- | --- | --- | --- | --- | --- |
|  | OR | 95% CI* | OR | 95% CI* | OR | 95% CI* |
| Age |  |  |  |  |  |  |
| 60-74 years | 1 |  |  |  |  |  |
| ≥75 years | 3.76 | 2.54-5.57 |  |  |  |  |
| Sex |  |  |  |  |  |  |
| Male | 1 |  | 1 |  | 1 |  |
| Female | 2.00 | 1.37-2.91 | 1.34 | 0.86-2.08 | 5.04 | 2.48-10.25 |
| Education |  |  |  |  |  |  |
| ≥ 7 years of education | 1 |  | 1 |  | 1 |  |
| 0-6 years of education | 1.17 | 0.70-1.96 | 1.11 | 0.64-1.91 | 2.28 | 0.54-9.63 |
| Living area |  |  |  |  |  |  |
| Suburban | 1 |  | 1 |  | 1 |  |
| Rural | 2.19 | 1.50-3.19 | 2.21 | 1.42-3.46 | 2.09 | 1.00-4.37 |
| Income |  |  |  |  |  |  |
| ≥ minimum wage | 1 |  | 1 |  | 1 |  |
| <minimum wage | 1.24 | 0.65-2.36 | 1.29 | 0.60-2.79 | 1.39 | 0.39-4.93 |
| No Income | 1.69 | 0.97-2.94 | 2.12 | 1.07-4.23 | 1.39 | 0.48-3.92 |
| Marital Status |  |  |  |  |  |  |
| Still married | 1 |  | 1 |  | 1 |  |
| Single (never married/divorce) | 1.80 | 1.18-2.77 | 1.75 | 1.05-2.89 | 1.72 | 0.69-4.26 |
| Occupational class |  |  |  |  |  |  |
| Professional | 1 |  |  |  | 1 |  |
| Not working | 2.12 | 1.11-4.02 | 2.87 | 1.33-6.22 | 1.01 | 0.28-3.66 |
| Laborer | 1.84 | 0.88-3.82 | 2.76 | 1.14-6.46 | 0.62 | 0.15-2.59 |
| Housewife | 1.78 | 0.70-4.50 | 2.15 | 0.69-6.67 | 1.28 | 0.18-8.88 |
| Hypertension | 1.11 | 0.77-1.60 | 1.03 | 0.68-1.60 | 1.42 | 0.68-2.98 |
| Stroke | 3.77 | 1.19-11.84 | 3.06 | 0.80-11.73 | 8.18 | 0.71-93.37 |
| Diabetes | 1.00 | 0.51-1.98 | 1.21 | 0.53-2.80 | 0.68 | 0.22-2.14 |
| Triglyceride > 200mg/dl (n, %) | 0.84 | 0.34-2.04 | 0.70 | 0.26-1.90 | 1.63 | 0.12-22.08 |
| HDL < 40mg/dl (n, %) | 0.65 | 0.33-1.29 | 0.85 | 0.41-1.77 | 0.22 | 0.64-1.69 |
| BMI Characteristics |  |  |  |  |  |  |
| Normal | 1 |  | 1 |  | 1 |  |
| Underweight | 1.30 | 0.80-2.19 | 2.04 | 1.20-3.48 | 1.12 | 0.49-2.53 |
| Overweight | 0.34 | 0.16-0.69 | 0.45 | 0.22-0.91 | 0.15 | 0.02-0.85 |
| Obese | 0.71 | 0.21-2.35 | 0.53 | 0.15-1.89 | 0.88 | 0.04-18.03 |
| Smoking status | 1.16 | 0.54-2.47 | 1.01 | 0.46-2.20 | 5.17 | 0.62-43.86 |
| Depression | 2.21 | 1.04-4.71 | 3.75 | 1.63-8.66 | 1.06 | 0.32-3.54 |
| Leisure activities |  |  |  |  |  |  |
| Intellectually less active | 2.97 | 1.81-4.89 | 2.96 | 1.70-5.19 | 3.13 | 1.02-9.60 |
| Socially less active | 1.74 | 1.19-2.52 | 1.79 | 1.16-2.77 | 1.56 | 0.72-3.35 |
| Recreationally less active | 2.49 | 1.69-3.67 | 2.43 | 1.56-3.79 | 2.4 | 1.07-5.58 |
| Physically less active | 2.72 | 1.35-5.50 | 2.40 | 1.11-5.18 | 0.52 | 0.28-0.94 |
| Less active (Total leisure activity) | 2.72 | 1.86-3.99 | 2.67 | 1.71-4.16 | 2.89 | 1.29-6.15 |
| Dietary Intake |  |  |  |  |  |  |
| Non-Frequent Carbohydrate intake | 1.36 | 0.95-1.96 | 1.68 | 1.08-2.60 | 0.71 | 0.34-1.49 |
| Non-Frequent Protein intake | 1.13 | 0.79-1.61 | 1.37 | 0.90-2.09 | 0.60 | 0.29-1.25 |
| Non-Frequent Fruit intake | 2.08 | 1.25-3.47 | 2.15 | 1.18-3.92 | 2.34 | 0.83-6.55 |
| Non-Frequent Vegetable intake | 1.09 | 0.76-1.57 | 1.17 | 0.76-1.81 | 0.90 | 0.44-1.83 |

**Table 9.** Univariable associations between potential risk factors and dementia in the total sample (n=686) and stratified by age (With missing data handled by mean substitution)

*OR odd ratio; 95%CI 95% confidence interval *Associations were adjusted for sex and education.*

**Table 10.** Multivariable models for the total sample (n=686) and stratified by age (With missing data handled by mean substitution)

|  | Total Sample | | 60-74 years | | ≥75 years | |
| --- | --- | --- | --- | --- | --- | --- |
|  | OR | 95% CI* | OR | 95% CI* | OR | 95% CI* |
| Age |  |  |  |  |  |  |
| 60-74 years | 1 |  |  |  |  |  |
| ≥75 years | 3.07 | 1.94-4.47 |  |  |  |  |
| Sex |  |  |  |  |  |  |
| Male | 1 |  | 1 |  | 1 |  |
| Female | 1.56 | 0.92-2.64 | 1.33 | 0.79-2.24 | 4.63 | 1.68-12.77 |
| Education |  |  |  |  |  |  |
| ≥ 7 years of education | 1 |  | 1 |  | 1 |  |
| 0-6 years of education | 0.49 | 0.26-0.92 | 0.56 | 0.30-1.04 | 1.20 | 0.24-5.93 |
| Living area |  |  |  |  |  |  |
| Suburban | 1 |  | 1 |  | 1 |  |
| Rural | 1.90 | 1.23-2.93 | 1.59 | 0.97-2.59 | 2.52 | 1.12-5.66 |
| Income |  |  |  |  |  |  |
| ≥ minimum wage | 1 |  | 1 |  | 1 |  |
| <minimum wage | 0.95 | 0.47-1.31 | 0.93 | 0.40-2.13 | 0.93 | 0.40-2.13 |
| No Income | 1.73 | 0.96-3.13 | 2.01 | 0.98-4.10 | 1.43 | 0.43-4.71 |
| Marital Status |  |  |  |  |  |  |
| Still married | 1 |  | 1 |  | 1 |  |
| Single (never married/divorce) | 1.07 | 1.03-2.86 | 0.90 | 0.84-0.95 | 1.82 | 0.67-4.93 |
| Occupational class |  |  |  |  |  |  |
| Professional | 1 |  | 1 |  | 1 |  |
| Not working | 1.93 | 0.93-3.89 | 2.28 | 1.00-5.22 | 1.17 | 0.27-5.13 |
| Laborer | 1.65 | 0.74-3.64 | 2.30 | 0.89-5.92 | 0.22 | 0.27-1.65 |
| Housewife | 1.96 | 0.64-5.62 | 2.26 | 0.66-7.71 | 01.26 | 0.09-17.11 |
| Hypertension | 1.11 | 0.74-1.65 | 1.07 | 0.67-1.71 | 1.19 | 0.51-2.73 |
| Stroke | 3.25 | 0.92-11.44 | 2.08 | 0.43-10.11 | 10.12 | 0.85-120.47 |
| Diabetes | 1.24 | 0.53-2.86 | 1.34 | 0.53-3.41 | 0.71 | 0.21-2.33 |
| Triglyceride >200mg/dl | 1.21 | 0.46-3.17 | 0.99 | 0.34-2.85 | 4.01 | 0.11-145.04 |
| HDL <40mg/dl | 0.60 | 0.29-1.12 | 0.77 | 0.36-1.67 | 0.20 | 0.02-1.93 |
| BMI Characteristics |  |  |  |  |  |  |
| Normal | 1 |  | 1 |  | 1 |  |
| Underweight | 1.30 | 0.80-2.19 | 1.59 | 0.89-2.84 | 1.10 | 0.440-2.77 |
| Overweight | 0.34 | 0.16-0.69 | 0.38 | 0.17-0.82 | 0.15 | 0.02-1.02 |
| Obese | 0.71 | 0.21-2.35 | 0.73 | 0.19-2.70 | 0.45 | 0.02-8.34 |
| Smoking status | 1.16 | 0.54-2.47 | 0.86 | 0.37-1.98 | 1.14 | 0.31-4.13 |
| Depression | 2.21 | 1.04-4.71 | 3.13 | 1.25-7.85 | 0.69 | 0.16-2.90 |
| Leisure activities |  |  |  |  |  |  |
| Intellectually less active | 3.06 | 1.79-5.22 | 3.14 | 1.74-5.69 | 2.39 | 0.68-8.39 |
| Socially less active | 1.63 | 1.08-2.45 | 1.76 | 1.10-2.82 | 1.20 | 1.03-1.32 |
| Recreationally less active | 2.32 | 1.52-3.55 | 2.27 | 1.40-3.67 | 2.11 | 0.83-5.35 |
| Physically less active | 2.35 | 1.14-4.83 | 2.08 | 0.94-4.60 | 2.83 | 0.49-16.26 |
| Less active (total leisure activity) | 2.46 | 1.62-3.72 | 2.57 | 1.59-4.14 | 1.87 | 0.76-4.55 |
| Dietary Intake |  |  |  |  |  |  |
| Non-frequent carbohydrate intake | 1.28 | 0.86-1.90 | 1.51 | 0.94-2.41 | 0.79 | 0.35-1.75 |
| Non-frequent protein intake | 0.88 | 0.59-1.30 | 1.07 | 0.67-1.71 | 0.48 | 0.21-1.09 |
| Non-frequent fruit intake | 1.97 | 1.14-3.38 | 1.96 | 1.04-3.70 | 2.55 | 0.83-7.77 |
| Non-frequent vegetable intake | 0.98 | 0.66-1.46 | 1.03 | 0.64-1.65 | 0.79 | 0.38-1.90 |

*OR odd ratio; 95%CI 95% confidence interval. Multivariable models include age, sex, education, plus all risk factors with ORs<0.75 or >1.40 in the univariable models (Table 9).*
